# Age-related impairment of navigation and strategy in virtual star maze

**DOI:** 10.1186/s12877-021-02034-y

**Published:** 2021-02-05

**Authors:** Jia-Xin Zhang, Lin Wang, Hai-Yan Hou, Chun-Lin Yue, Liang Wang, Hui-Jie Li

**Affiliations:** 1 CAS Key Laboratory of Behavioral Science, Institute of Psychology, 16 Lincui Road, Beijing, 100101 China; 2 Department of Psychology, University of Chinese Academy of Sciences, Beijing, 100049 China; 3 CAS Key Laboratory of Mental Health, Institute of Psychology, 16 Lincui Road, Beijing, 100101 China; 4 College of Physical Education and Sport Science, Soochow University, Suzhou, 215021 China

**Keywords:** Aging, Virtual environment, Star maze, Navigation, Egocentric strategy

## Abstract

**Background:**

Although it is well known that aging impairs navigation performance, the underlying mechanisms remain largely unknown. Egocentric strategy requires navigators to remember a series of body-turns without relying on the relationship between environmental cues. Previous study suggested that the egocentric strategy, compared with non-egocentric strategy, was relatively unimpaired during aging. In this study, we aimed to examine strategy use during virtual navigation task and the underlying cognitive supporting mechanisms in older adults.

**Methods:**

Thirty young adults and thirty-one older adults were recruited from the local community. This study adapted star maze paradigm using non-immersive virtual environment. Participants moved freely in a star maze with adequate landmarks, and were requested to find a fixed destination. After 9 learning trials, participants were probed in the same virtual star maze but with no salient landmarks. Participants were classified as egocentric or non-egocentric strategy group according to their response in the probe trial.

**Results:**

The results revealed that older adults adopting egocentric strategy completed the navigation task as accurate as young adults, whereas older adults using non-egocentric strategy completed the navigation task with more detours and lower accuracy. The relatively well-maintained egocentric strategy in older adults was related to better visuo-spatial ability.

**Conclusions:**

Visuo-spatial ability might play an important role in navigation accuracy and navigation strategy of older adults. This study demonstrated the potential value of the virtual star maze in evaluating navigation strategy and visuo-spatial ability in older adults.

**Supplementary Information:**

The online version contains supplementary material available at 10.1186/s12877-021-02034-y.

## Background

Navigation means finding and maintaining a route in a familiar or unfamiliar environment [1]. It is one of the fundamental cognitive functions that decline the most with increasing age [2]. Empirical studies have found that navigation is vulnerable to aging [3]. Compared with young adults, older adults tend to commit more errors and take longer in solving navigation tasks [4].

Many studies have found that there are two main strategies during navigation: egocentric strategy and allocentric strategy [5–7]. Allocentric strategy relies on a world-centred representation, whereas egocentric strategy is based on a self-centered (i.e., body-centred, a series of left and right turns) representation [7, 8]. Specifically, allocentric strategy is based on a “map-like” representation, which enables the navigators to find a detour or reach a destination from different starting point [9, 10]. In contrast, egocentric strategy is based on a series of association of stimulus and responses (idiothetic information such as body turns and vestibular sense) and allows individuals to navigate in a fixed route [7, 8]. This strategy enables individuals to navigate from a fixed origin to a fixed destination through the same route [9]. Beyond the aforementioned two strategies, guidance strategy was also involved in human navigation. This strategy requires the navigators to remember a salient landmark closer to the destination. To find the destination, navigators keep moving towards the landmark instead of recalling the “map-like” representation or a series of body-turns [6, 11]. Guidance strategy poses little pressure on cognitive load. In contrast, the allocentric strategy requires the navigator to remember the spatial relationships between landmarks and form a cognitive map, which needs more cognitive load. Spatial memory task involving egocentric and allocentric strategy may be a sensitive tool to detect the cognitive decline during aging [12]. In age-related studies, previous studies have found the egocentric strategy is relatively preserved in older adults, while allocentric strategy and other strategies experience decline [2, 9, 13, 14]. According to the retrogenesis theory, abilities acquired later in life are more vulnerable to aging and are the first to deteriorate [15]. As a more fundamental representation, egocentric strategy is acquired earlier, and is more rooted and well-preserved in older adults [16]. Several studies reported that the visuo-spatial deficits might help discriminate mild cognitive impairment (MCI) from dementia in elderly population [17–19]. Using different 2-dimensional visuoconstructive tasks, previous study found that the real world navigation was selectively related with the visuo-spatial memory [20]. Though the visuo-spatial working memory correlated with the navigation performance both in young and older adults, visuo-spatial working memory performance decreased with age [21]. Therefore, visuo-spatial ability may support the strategy use and navigation performance in older adults. Although it is widely recognized that successful navigation relies on both egocentric and non-egocentric strategies [22], the neurobiological underpinnings are different. The non-egocentric strategy relies mainly on hippocampus and the retrosplenial cortex, while egocentric strategy is mainly dependent on caudate nucleus, medial parietal lobe, and posterior parietal area [13]. Hippocampus and retrosplenial cortex experience rapid structural and functional decline during aging [23–27], leading to more declines in non-egocentric navigations. Due to the difficulties in construction and storage spatial information, non-egocentric navigation deficit was considered as a possible biomarker for early AD diagnosis [28, 29]. Due to navigation has fewer verbal, cultural and educational biases, exploring the age-related egocentric and non-egocentric navigation decline could be helpful to find the cognitive fingerprints of AD and cognitive intervention outcome measures [30]. Recent studies revealed that a successful navigation primarily relies on the capacity to flexibly choose the appropriate strategy depending on the task demands rather than using one specific strategy [13, 31]. Therefore, though some evidence suggests that the egocentric strategy is well preserved in older adults, the navigation performance is still impaired if they fail to choose the appropriate strategy. A virtual environment study has already confirmed the capacity to choose both allocentric and egocentric strategy in young adults [7]. In contrast, older adults appear to have a deficit in strategy choosing and are more likely to choose the inappropriate strategy across different studies [32, 33].

Visuo-spatial ability is typically assessed using paper-and-pencil tasks such as complex figure task and clock drawing test [3]. These tasks were designed to evaluate the basis spatial abilities, for instance mental rotation and spatial memory, which are moderately or weakly correlated with spatial memory or navigation in the real world [3, 34, 35]. Recently, a study compared walking and non-walking space task in an equivalent virtual environment [36]. It shows that walking space task is much easier for participants to search different viewpoints to get themselves familiar with the environment and locate the targets, whereas the non-walking space task may demand extra cognitive effort. Therefore, an environment in which participants can move and rotate freely should be more compatible for older adults and sensitive to the effect of aging [37]. Given that the real-world study is relatively complex, costly, and difficult to control, virtual environment becomes an attractive approach to investigate navigation [38–40]. Previous studies have found that navigators could spontaneously form two different spatial representations by passively watching the simple optic flow [41]. By contrast, the non-immersive virtual environment could provide more abundant environment details and vividly simulate the real world. A previous study reported a strong correlation between virtual environment and real-world navigation performance [42], indicating the applicability of virtual environment in navigation studies. Moreover, non-immersive virtual environment could be used in diagnosis of the spatial navigation disorders [43]. Contrarily, the paper-pencil tests only showed partial correlation with the real space tests [35]. Recently, the non-immersive virtual environment technique with elaborately designed scenes has been widely used in navigation tasks, including the Morris Water maze [44], the Eight-Arm maze [31, 45, 46], and the star maze [7, 47]. However, it is worth noting that a virtual environment navigation paradigm may be good at distinguishing navigation strategies but weak in evaluating navigation performance (e.g., navigational speed, distance travelled), or vice versa [41, 46]. The virtual star maze may be a trade-off for evaluating both navigation performance and navigation strategy [48]. In the virtual star maze task, participants navigate freely in a pentagram-shaped virtual environment and the task is always to find a fixed destination. The latency, total degree rotated and distance travelled are recorded as navigation performance. In addition, landmarks may be removed or changed to detect the strategy [7, 28, 48]. Previous studies have already applied the virtual star maze in healthy children [6] and young adults [7]. Moreover, virtual star maze might provide a selective behavioural marker for AD [28]. Therefore, in healthy aging, the virtual star maze is considered as an ideal paradigm to evaluate age-related changes both in navigation performance and strategy.

In the current study, we aimed to use the virtual environment technique to examine strategy use and the supporting cognitive mechanism in older adults. We expected that older adults preferred to use egocentric strategy, and the success of using egocentric strategy in older adults was related with visuo-spatial ability.

## Methods

### Participants

Thirty-one young adults (15 males and 16 females; mean age: 22.68 ± 3.07) and thirty-two older adults (14 males and 18 females; mean age: 66.91 ± 5.27) were recruited using notices distributed at local universities or in the community or by word of mouth. Inclusion criteria of participants were as follows: 1) no history of neurological or psychiatric diseases based on self-report; 2) 6 years or more of education; 3) a score of 24 or higher on the Mini-Mental State Examination (MMSE) [49]; 4) for young adult group, the age should be within the range from 18 to 30, for older adult group, the age should be over 55. The study was approved by the local ethics committee of the Institute of Psychology, Chinese Academy of Sciences (IPCAS). Written informed consent was obtained from all participants in accordance with the Declaration of Helsinki prior to the study.

Participants were asked about their frequency of computer and virtual environment use. For instance, ‘How often do you use computers? 1 for never, 2 for occasionally, 3 for often’.

### Neuropsychological tests

All participants completed neuropsychological tests, including the Multifactorial Memory Questionnaire (MMQ) [50], the digit span tests and block design. MMQ was used for general memory status estimation; it included three subtests: memory content, memory ability and memory strategy. The digit span tests were selected from the Wechsler Adult Intelligence Scale (WAIS) [51], including the forward and backward version. The block design test, also selected from the WAIS-IV, mainly evaluates visuo-spatial ability. The order of neuropsychological tests was counterbalanced among participants.

### The virtual star maze paradigm

During the entire duration of the following virtual environment, participants were comfortably seated in front of the computer screen and moved the joystick (Sony DualShock 4) freely in a first-person view. Prior to the virtual star maze task, participants went through a joystick-familiarity task. Moreover, participants were asked to move along the boundary of a yellow square different from the star maze task to help familiarize with the joystick. Movement in virtual environment was achieved by pushing the joystick forward and pulling it backward, and horizontal rotation was achieved by pushing the joystick left and right. Young adults spent an average of 5 min and older adults an average of 15 min in this joystick training. After participants declared their confidence to complete the task, the joystick training ended and the star maze task began.

**Fig. 1:**
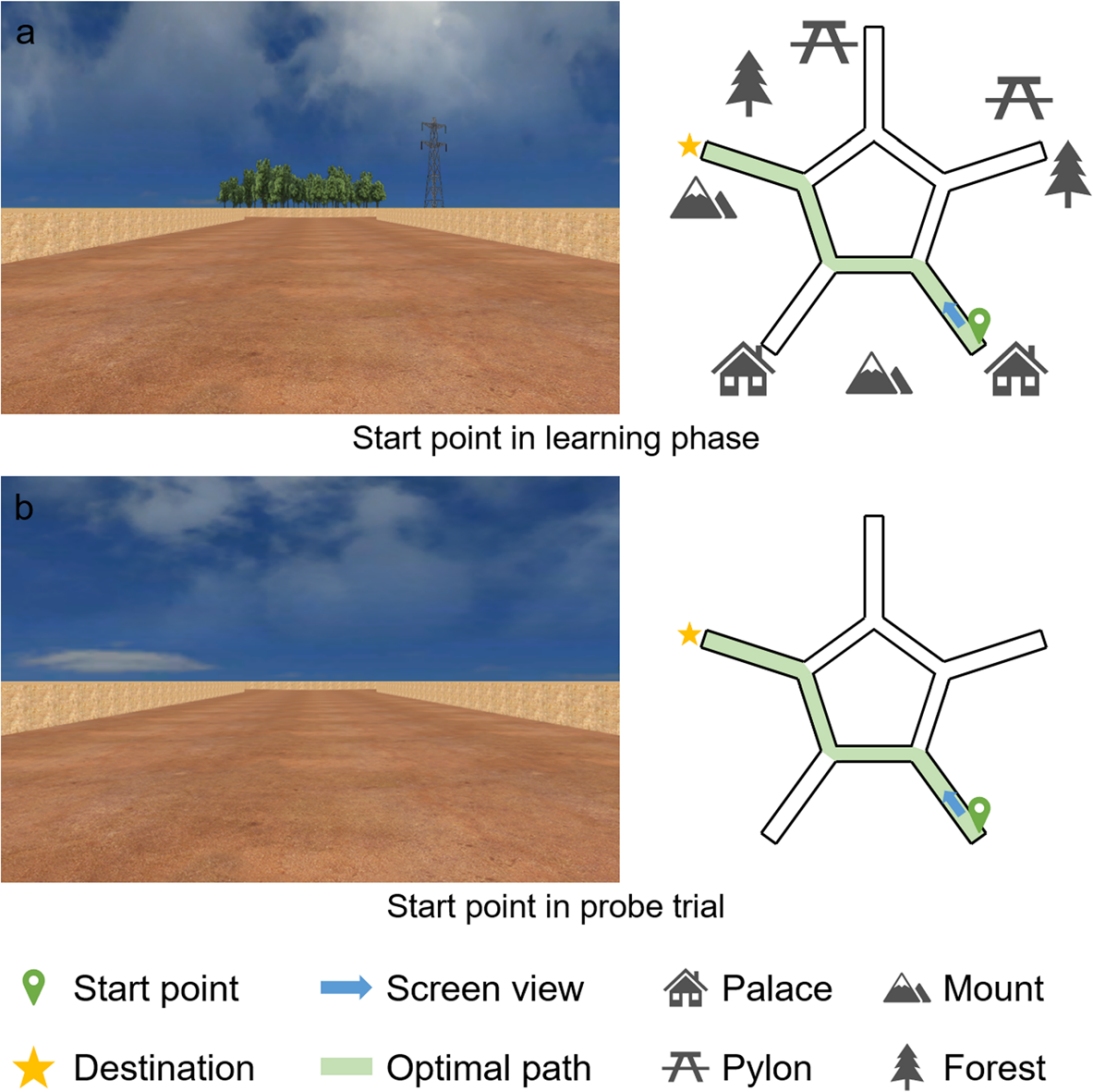
Screenshots and planforms of the virtual star maze. The green alleys in the right planforms represent the optimal path from the start point to the destination. The blue arrow shows the location and facing direction of the left screenshot. **a**. The left picture corresponds to the screenshot when participants were at the start point of each learning trial. The right picture presents all the surrounding landmarks, start point and destination. **b**. All the landmarks were absent in the probe trial. Participants began from the same start point with the same facing direction but with no salient landmarks

The human virtual star maze is similar to the well-defined rodent navigation paradigm. Current study adapted it from previous virtual star maze research in older adults and made a few changes [28]. Compare to the previous study that consisted of 11 learning trials, this study used 9 learning trials to reduce the occurrence of the dizziness. The 3-dimensional model of the star maze was designed by Sketchup (https://3dwarehouse.sketchup.com) and the landmarks were made by Maya (https://www.autodesk.com/products/maya). Then they were integrated into a virtual environment paradigm developed using the WorldViz Vizard 4.0 software (WorldViz LLC, http://www.worldviz.com). The maze comprised five central alleys (for each alley 16.38 virtual meter, vm) forming a pentagon and five alleys (37.5 vm for each alley) radiating from the angle of the central pentagon. The wall height was 2 vm and height of the virtual camera was set to 1 vm (Fig. 1). Participants used a joystick to freely move around at a constant speed (8 vm/s) and rotate (95°/s) in all the alleys. To eliminate the usage of guidance strategy, two similar distal landmarks were presented.

The virtual star maze task included two phases: the learning phase and the probe phase.

### Learning phase

Before the start of the entire experiment, participants were informed that the goal of the virtual environment task was to find and maintain a location, which would always be located at the same place during the following tests. They should always reach this same destination during the entire study. This destination is invisible, participants should find it according to the instructions and tasks during exploring the entire virtual environment in the following trials. Once reached, sparking fireworks would be shown to provide a reward and signal the beginning of the next trial. If they learnt the destination, they should always take the optimal route to the destination, which means they should not enter any irrelevant alleys.

Prior to the learning phase, all participants went through at least two practice trials to ensure they learned the task and remembered the destination. The goal of the practice trial is the same as the learning phase, and the participants need to reach the same destination. However, in the practice trials, if the participants fail to find the destination within 90 s, they will be teleported to the right alley facing toward the destination, aiming to help participants familiarize themselves with the task. While in the learning trials, the next trial will restart if the participants cannot find the destination. In the practice trials, if participants thought they were able to locate the destination after two practice trials, then the formal learning phase would begin; otherwise, participants were provided with additional practice trials.

The learning phase was designed to evaluate navigation performance and learning effect. In each trial, participants would always start from the same place with same facing direction to the same destination (Fig. 1a, indicated by the blue arrow on the left-hand pictures). Instructions were designed to avoid any words involving view, route, circumstance, etc., which excluded bias from any strategy. Participants were provided with 9 learning trials. Each learning trial had a 90-s time limitation; if participants failed to reach the destination within 90 s, the trial terminated immediately and the next trial began. In the learning phase, participants could reach the destination by remembering the sequence of body turns of each Y-shaped intersection (egocentric strategy) or other strategies.

A computer automatically collected the location every 20 milliseconds, as well as the facing direction related to the start point. The total travel distance and time of navigation were also recorded for each learning trial. The total distance travelled was expressed in vm and the time in milliseconds. To evaluate the navigation performance of each participant, the following parameters were calculated for the 9 learning trials: speed, distance error, rotation, percentage of successful people and percentage of successful trials. Notably, in current study, speed was considered as measurement of navigation efficiency, whereas the distance error, rotation, percentage of successful people and percentage of successful trials were considered as measurement of the navigation accuracy.

Speed was calculated by dividing total travel distance by total time and was expressed in virtual length units per second (Formula 1). Speed here included stopping time and therefore was considered a factor in navigation efficiency.

1 $$ \mathrm{Speed}\kern0.5em \left(\mathrm{vm}/\mathrm{s}\right)=\frac{\mathrm{total}\kern0.5em \mathrm{travel}\kern0.5em \mathrm{distance}\kern0.5em }{\mathrm{total}\kern0.5em \mathrm{time}} $$

Rotation corresponded to the total degrees each participant rotated. The more degrees participants rotated to complete the task, the less accurate they were. This parameter was calculated by summing up the absolute value of the facing direction changes between both adjacent time points (Formula 2).

2 $$ \mathrm{Rotation}\left({}^{\circ}\right)=\sum \mid {\mathrm{facing}\ \mathrm{direction}}_{\mathrm{tn}}-{\mathrm{facing}\ \mathrm{direction}}_{\mathrm{tn}-1}\mid $$

Distance error was calculated by subtracting the optimal path distance from the total travel distance. To compare distance error between individuals, we further divided this result by the optimum path distance (Formula 3). Participants who travelled the optimal path exactly scored 0% in distance error, whereas a detour would increase distance error.

3 $$ \mathrm{Distance}\kern0.5em \mathrm{error}\kern0.5em \left(\%\right)=\frac{\mathrm{navigation}\kern0.5em \mathrm{distance}-\mathrm{optimum}\kern0.5em \mathrm{path}\kern0.5em \mathrm{distance}}{\mathrm{optimum}\kern0.5em \mathrm{path}\kern0.5em \mathrm{distance}}\times 100\% $$

Moreover, each trial participants made was recorded either 1 for a successful navigation or 0 for fail. For each learning trial, we calculated the percentage of successful people among young and older adults separately (Formula 4). And for each participant, we calculated the percentage of successful trials over the 9 learning trials (Formula 5).

4 $$ \mathrm{Successful}\kern0.5em \mathrm{people}\kern0.5em \left(\%\right)=\frac{\mathrm{number}\kern0.5em \mathrm{of}\kern0.5em \mathrm{successful}\kern0.5em \mathrm{participants}\kern0.5em \mathrm{in}\kern0.5em \mathrm{each}\kern0.5em \mathrm{group}}{\mathrm{number}\kern0.5em \mathrm{of}\kern0.5em \mathrm{the}\kern0.5em \mathrm{participants}\kern0.5em \mathrm{in}\kern0.5em \mathrm{each}\kern0.5em \mathrm{group}}\times 100\% $$

5 $$ \mathrm{Successful}\kern0.5em \mathrm{trials}\kern0.5em \left(\%\right)=\frac{\mathrm{number}\kern0.5em \mathrm{of}\kern0.5em \mathrm{successful}\kern0.5em \mathrm{trials}\kern0.5em }{9\kern0.5em \mathrm{learning}\kern0.5em \mathrm{trials}}\times 100\% $$

### Probe phase

After the 9 learning trials, participants had to finish one probe trial within 90 s. The probe trial shared the same maze structure as the learning phase but with no salient landmarks (i.e., all distal cues were removed). For the volunteers, the probe trial just like the tenth learning trial, the existence of the probe trial was not mentioned in advance. Even the total number of trials were not informed before the experiment, volunteers were only been told the experiments consists of dozens of trials, the program will stop automatically when it ends.

The probe trial was designed to distinguish the strategy used and the navigation performance in probe trial was not analysed to avoid circular reasoning. The absence of distal landmarks made strategies relying on the landmarks invalid, including allocentric or guidance strategy. In contrast, egocentric strategy by remembering a sequence of body turns was unaffected. In consequence, participants were further divided into two strategy groups according to the probe trial. Those participants who reached the destination directly in the probe trial (did not enter any irrelevant alleys other than optimal path) were classified as egocentric strategy users (by remembering a series of body turns), whereas the rest of the participants who failed to perform a direct trial were classified as non-egocentric strategy users. The major difference between two strategy groups is if the participants could use the appropriate and efficient strategy when the environment and task quest changed. And then we looked back the learning phase, whether those older adults who were able to use an egocentric strategy in the probe trial also navigated much more efficient. Therefore, in the currents study, there were four subgroups: egocentric older adults, non-egocentric older adults, egocentric young adults and non-egocentric young adults.

### Data analysis

Demographics were analyzed using independent sample t tests for continuous variables and chi-square tests for categorical variables.

Three-way repeated-measure ANOVA was carried out for speed, rotation, distance error and percentage of successful trials separately, with age (young adults, older adults) and strategy (egocentric, non-egocentric) as between factors, and learning trials (9 learning trials) as within factor. Because of large rotation, 3 young adults (2 males and 1 female) exceeded three-standard deviations (SDs) from their group in the 6th and 7th learning trials, and were excluded from the repeated-measure ANOVAs and subsequent simple effect tests.

Pearson correlations among navigation performance during the learning phase and neuropsychological tests were applied in young and older adults separately. Moreover, to further explore whether the two strategy groups in older adults differed in WAIS block design and digit span, independent sample t tests were applied.

Furthermore, to validate the discrimination of the probe trial, we performed an additional analysis. We administered paired sample t tests between the 9th learning trial and the probe trial in egocentric strategy users and non-egocentric strategy users, respectively.

## Results

### Demographics

Two older adults were excluded for not completing the entire virtual environment task (one male and one female older adult). The final sample comprised of thirty-one young adults (15 males and 16 females; mean age: 22.68 ± 3.07) and thirty older adults (13 males and 17 females; mean age: 67.30 ± 5.10) (Table 1). Young and older adults were well matched in gender ratio, years of education, and MMSE. Moreover, older adults showed less exposure to computers and virtual environment than young adults. Young adults showed significantly better performance than older adults in WAIS-block design and digit span (both forward and backward versions). No significant differences were found between young and older adults in all three subtests of MMQ. Twenty-one young adults (67.7%) needed extra 1 to 4 practice trials (Mean = 1.10, SD = 1.06), and twenty-six older adults (86.7%) needed additional 1 to 7 practice trials (Mean = 2.57, SD = 1.93), the percentage did not show significantly differences (χ^2^ = 3.088, *p* = .079). However, older adults required significantly more practice trials than young adults (t = − 3.306, *p* = .002). Therefore, the number of practice trials was analyzed as the covariate in the following ANOVA.

**Table 1:** Demographic information and cognitive measures of young and older adults

	Young adults (*n* = 31)		Older adults (n = 30)		t
	M	SD	M	SD	
Age (years)	22.68	3.07	67.30	5.10	−41.24^***^
Education (years)	15.61	2.09	15.27	1.39	0.76
Computer exposure	1.23	0.43	1.73	0.78	−3.13^**^
Virtual environment exposure	2.48	0.72	2.97	0.18	−3.60^**^
MMSE	28.68	1.14	28.70	1.32	−0.07
MMQ-contentment	50.55	12.19	46.50	11.04	1.36
MMQ-ability	54.45	9.60	50.18	10.83	1.63
MMQ-strategy	33.94	15.08	30.42	14.57	0.94
WAIS block design	42.87	4.62	33.33	6.71	6.42^***^
Forward digit span	8.74	0.58	7.73	0.83	5.51^***^
Backward digit span	6.74	1.53	5.20	1.42	4.08^**^

*M* mean; *SD* standard deviation; *MMSE* Mini-Mental State Examination; *MMQ* Multifactorial Memory Questionnaire; *WAIS* Wechsler Adult Intelligence Scale-IV. ^**^ p < .01, ^***^ p < .001

### Strategy defined by the probe trial

According to the strategy discrimination criterion in probe trial, the current study comprised four groups: egocentric older adults, non-egocentric older adults, egocentric young adults and non-egocentric young adults. The final sample comprised of seven egocentric older adults (23.33%), twenty-three non-egocentric older adults (76.67%), seventeen egocentric young adults (54.84%) and fourteen non-egocentric young adults (45.16%), χ^2^ = 6.34, *p* = .012. Two strategy groups did not differ in age. Specifically, egocentric older adults age from 61 to 75 (mean age: 66.86 ± 5.640, 5 females and 2 males), non-egocentric older adults age from 57 to 78 (mean age: 67.43 ± 5.05, 12 females and 11 males), egocentric young adults age from 18 to 29 (mean age: 23.59 ± 3.14, 8 females and 9 males), non-egocentric young adults age from 18 to 28 (mean age: 21.57 ± 2.68, 8 females and 6 males) (Table 2).

**Table 2:** Demographic information of egocentric and non-egocentric strategy navigators in young and older adults

	Young adults (n = 31)		Older adults (n = 30)	
	Non-egocentric (*n* = 14)	Egocentric (*n* = 17)	Non-egocentric (*n* = 7)	Egocentric (*n* = 23)
Age (year)	21.57 ± 2.68	23.59 ± 3.14	67.43 ± 5.05	66.86 ± 5.64
Female/Male	8/6	8/9	12/11	5/2

**Fig. 2:**
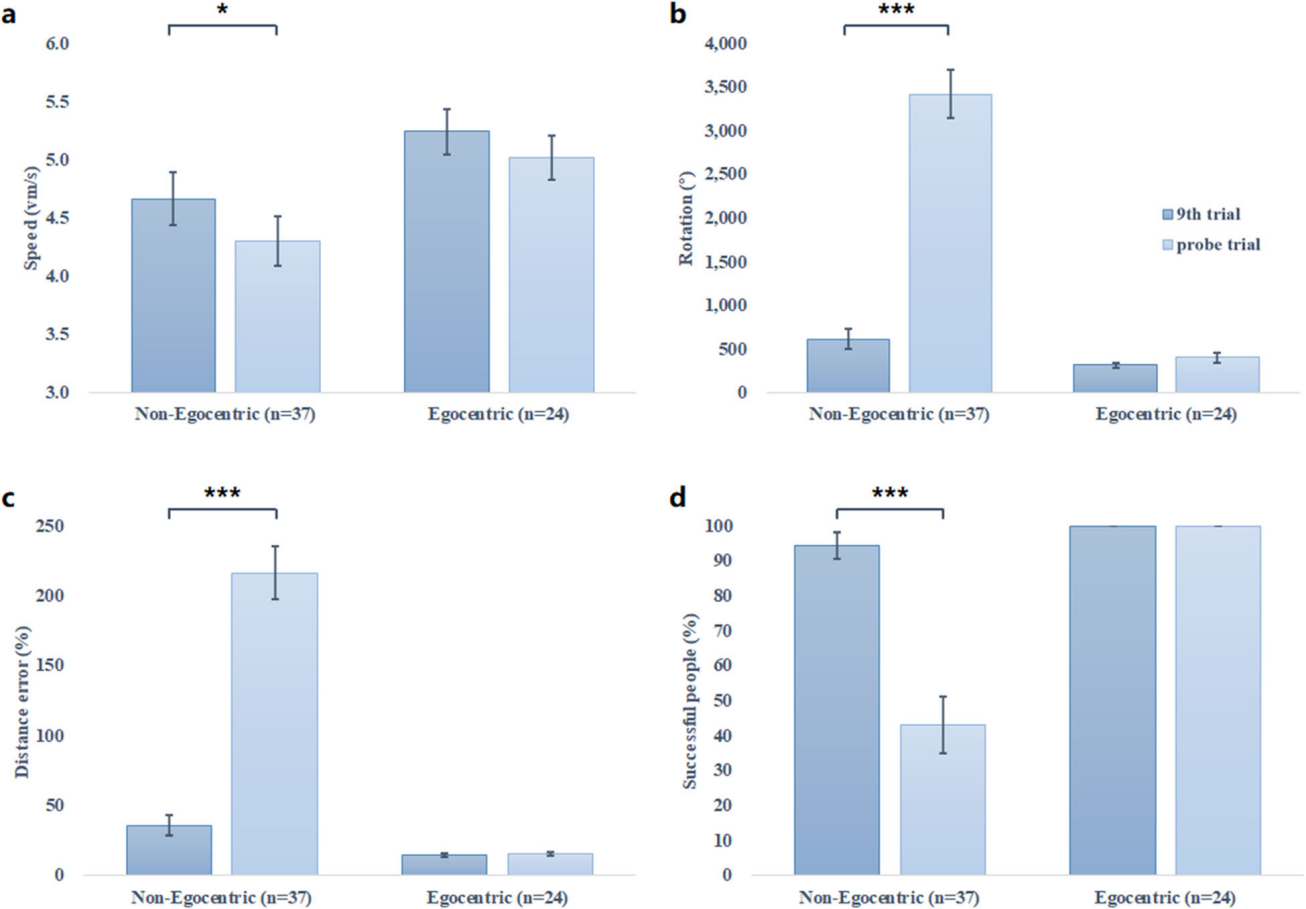
The navigation performance of non-egocentric (*n* = 37) and egocentric group (*n* = 24) in the 9th learning trial and probe trial. **a**. Only the non-egocentric strategy users navigated significantly more slowly in the probe trial than in the 9th learning trial. **b**. Only the non-egocentric strategy users performed more rotations in the probe trial than in the 9th learning trial. **c**. Non-egocentric strategy users navigated using more detours in the probe trial than in the 9th learning trial. **d**. The percentage of successful people in the non-egocentric group drops significantly from the 9th learning trial to the probe trial. ^*^p < .05, ^***^p < .001

Theoretically, the absence of distal landmarks in probe trial should only impair strategies relied on landmarks (i.e. allocentric strategy or guidance strategy), leaving the egocentric strategy unimpaired. As shown in Fig. 2, the performance of egocentric strategy users (including both young and older adults) did not differ between the 9th learning trial and the probe trial, whereas non-egocentric strategy users performed significantly worse in the probe trial than in the 9th learning trial, including slower navigation speed (t = 2.273, *p* = .029), more rotations (t = − 10.382, *p* < .001), higher distance error (t = − 9.113, *p* < .001) and lower percentage of successful people (t = 6.508, *p* < .001). This specific impairment of non-egocentric navigation from the 9th learning trial to the probe trial highly supported the strategy discrimination of the probe trial.

To be noted, we carefully checked all the trajectories of the participants. All participants classified into non-egocentric group entered more irrelevant alleys in probe trial compared with the 9th learning trial, except for 2 older adults, who entered the same number of irrelevant alleys during the probe trial and the 9th trial, indicating that the two older adults used “serial strategy” [52] by keeping turning left in all the intersections (Supplementary Fig. 1). This strategy needs less cognitive load and more like a trial-and-error procedure (e.g., I will certainly reach the destination if I keep turning left in each intersection), while egocentric strategy requires the navigators to combine a series of stimulus-response association (e.g., turn left in the first intersection and turn right when I see a forest). Therefore, these two older adults did not fully form an egocentric strategy according to their route and were classified as non-egocentric strategy users.

### Navigation performance in the learning phase

**Fig. 3:**
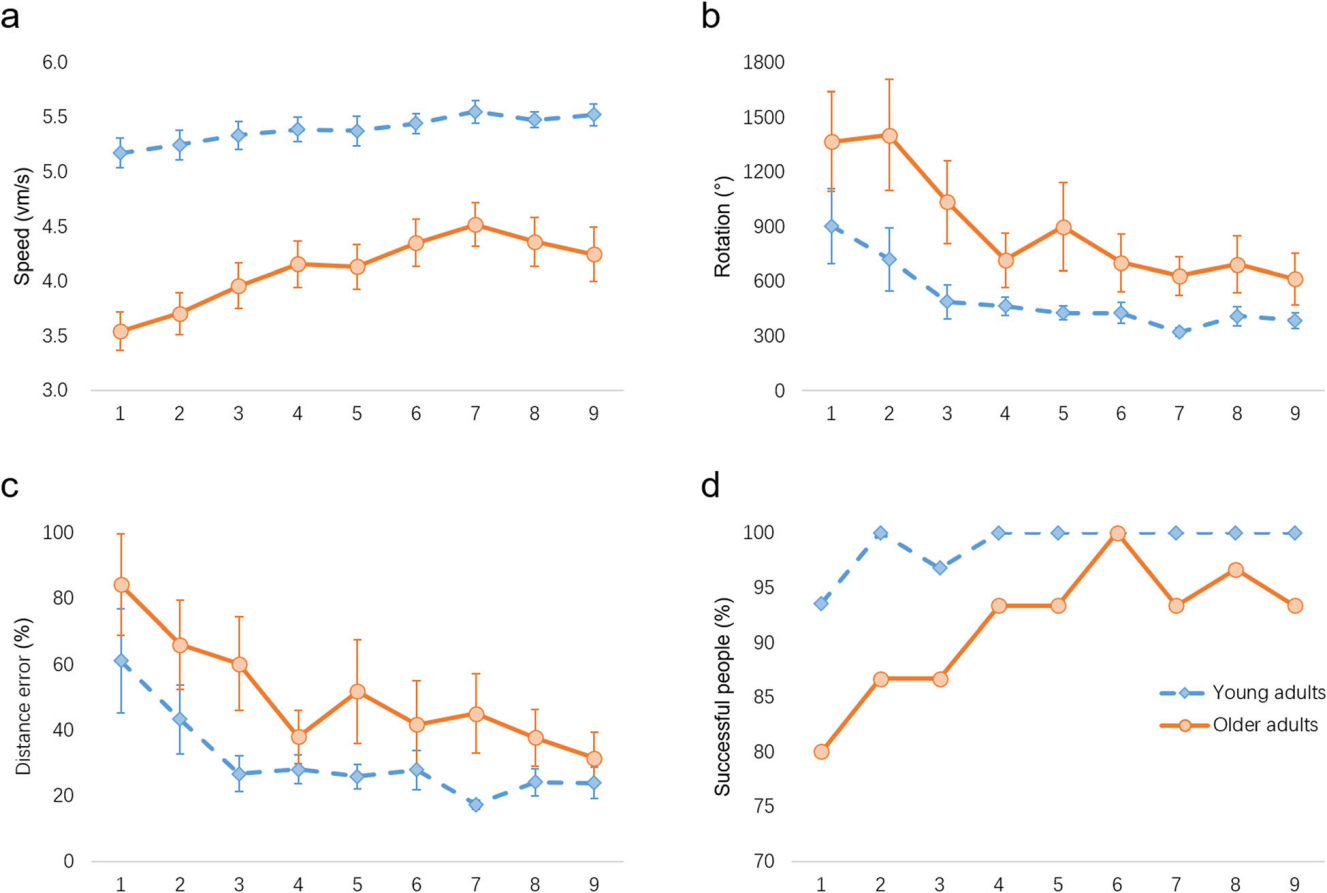
Navigation performance of young and older adults from the 1st to the 9th learning trial. **a**. Both young and older adults navigated faster with learning. **b**. Larger rotation indicated less accuracy during learning. With learning, both groups completed the task with less rotation. **c**. Larger distance error indicated more detour during learning, whereas 0% indicated the ideal path. **d**. Larger successful population indicated more successful participants in a group and 100% indicated that all members succeeded in reaching the destination in 90 s. 3 young adults were excluded in the 6th and 7th trials because they exceeded three standard deviations from the average in rotation. Error bars indicate mean ± SEM

According to the results of repeated-measure ANOVA (Table 3), the data did not fulfil the sphericity assumption (all ps < .001), we chose the Huynh-Feldt epsilon coefficient to adjust the degrees of freedom (ε = .654 for speed, ε = .655 for rotation, ε = .533 for distance error, ε = .619 for percentage of successful trials, separately). We found that the main effect of learning trials were significant in speed [F(5, 282) = 10.860, *p* < .001, η^2^ = .167], rotation [F(4, 195) = 2.535, *p* = .047, η^2^ = .045], and distance error [F(4, 230) = 2.796, *p* = .024, η^2^ = .049] (Fig. 3a, b, c). The main effect of learning trials did not reach significance in the percentage of successful trials [F(5, 268) = .627, *p* = .678, η^2^ = .011] (Fig. 3d). Given that the percentages of successful trials were close to 100% during the entire learning phase, the absence of learning effect may be the consequence of the ceiling effect.

**Table 3:** Repeated-measure ANOVA with age and strategy as between factors and trial as within factor (*n* = 58)

	Speed (vm/ second)	Rotation (°)	Distance error (%)	Successful trials (%)
Age				
F	25.906	2.711	2.235	3.584
p	< 0 .001^***^	0.105	0.141	0.064
Effect size (η^2^)	0.324	0.048	0.040	0.062
Strategy				
F	0.385	6.969	6.326	1.960
p	0.538	0.011^*^	0.015^*^	0.167
Effect size (η^2^)	0.007	0.114	0.105	0.035
Trial				
F	10.860	2.535	2.796	0.627
p	< 0 .001^***^	0.047^*^	0.024^*^	0.678
Effect size (η^2^)	0.167	0.045	0.049	0.011
Age × Strategy				
F	0.524	7.385	7.182	4.037
p	0.472	0.009^**^	0.010^**^	0.050^*^
Effect size (η^2^)	0.010	0.120	0.117	0.070
Age × Trial				
F	1.284	0.123	0.279	0.094
p	0.269	0.966	0.902	0.993
Effect size (η^2^)	0.023	0.002	0.005	0.002
Strategy × Trial				
F	0.525	0.150	0.304	0.600
p	0.765	0.952	0.885	0.698
Effect size (η^2^)	0.010	0.003	0.006	0.011
Age × Strategy × Trial				
F	1.136	1.024	1.160	1.321
p	0.342	0.392	0.330	0.256
Effect size (η^2^)	0.021	0.019	0.021	0.024

3 young adults (2 males and 1 female) were excluded from the repeated-measure ANOVA because of exceeding three-standard deviations in rotation. ^*^ p < .05, ^**^ p < .01, ^***^ p < .001

**Fig. 4:**
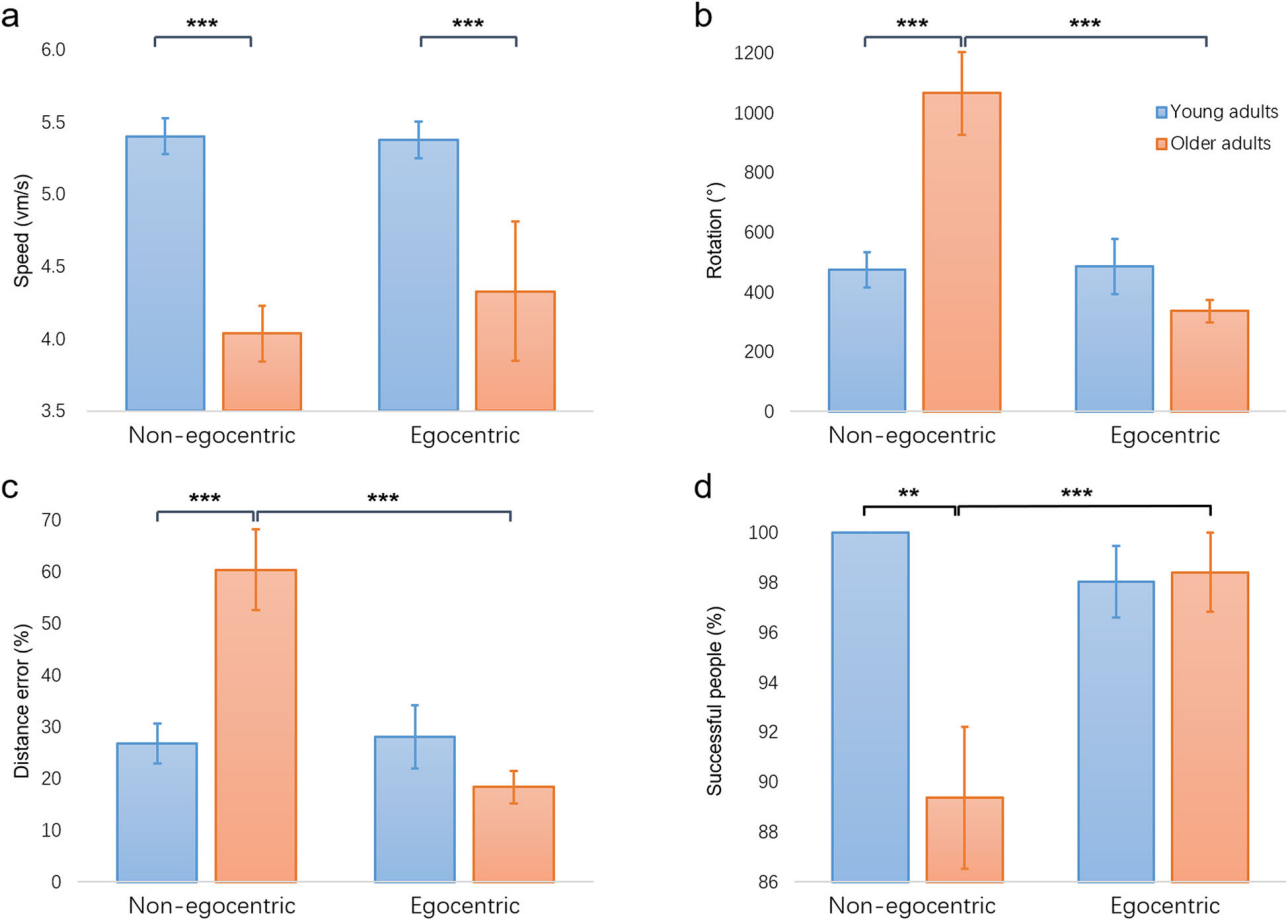
The average of navigation performance of both young and older adults in the learning phase. **a**. Older adults completed the star maze using a slower navigation speed during the entire learning phase. **b**. Non-egocentric older adults performed significantly more rotations than non-egocentric young adults and egocentric older adults in the learning phase. **c**. Non-egocentric older adults completed the task with more detours than non-egocentric young adults and egocentric older adults during the learning phase. **d**. The success rate of non-egocentric older adults during the learning phase was significantly lower than for non-egocentric young adults and egocentric older adults. Error bars indicate mean ± SEM. ^**^p < .01, ^***^p < .001

The main effect of age was significant in speed [F(1, 54) = 25.906, p < .001, η^2^ = .324]. Young adults navigated significantly faster than older adults (Fig. 4a). None of the other main effects of age reached significance, including rotation [F(1, 54) = 2.711, *p* = .105, η^2^ = .048], distance error [F(1, 54) = 2.235, *p* = .141, η^2^ = .040] and percentage of successful trials [F(1, 54) = 3.584, *p* = .064, η^2^ = .062].

The main effect of strategy was significant in rotation [F(1, 54) = 6.969, *p* = .011, η^2^ = .114] and distance error [F(1, 54) = 6.326, *p* = .015, η^2^ = .105]. Non-egocentric strategy users completed the virtual star maze task with more rotations and higher distance error. No significant effects were found in speed [F(1, 54) = .385, *p* = .538, η^2^ = .007] and percentage of successful trials [F(1, 54) = 1.960, *p* = .167, η^2^ = .035].

The interactions between age and strategy were significant in rotation [F(1, 54) = 7.385, *p* = .009, η^2^ = .120], distance error [F(1, 54) = 7.182, *p* = .010, η^2^ = .117] and percentage of successful trials [F(1, 54) = 4.037, *p* = .050, η^2^ = .070]. As shown in Fig. 4, all simple effect tests revealed similar results: the performances of non-egocentric older adults were significantly worse than those of egocentric older adults and non-egocentric young adults. Compared with egocentric older adults, non-egocentric older adults completed the task with more rotations [F(1, 57) = 20.81, *p* < .0011, η^2^ = 1.568], higher distance error [F(1, 57) = 19.35, p < .001, η^2^ = 1.574] and lower percentage of successful trials [F(1, 57) = 10.49, *p* = .002, η^2^ = .910], suggesting that the non-egocentric strategy was less accurate than the egocentric strategy in older adults. Compared with non-egocentric young adults, non-egocentric older adults completed the task with more rotations [F(1, 57) = 15.49, p < .001, η^2^ = 1.241], higher distance error [F(1, 57) = 14.31, p < .001], η^2^ = 1.226 and lower percentage of successful trials [F(1, 57) = 11.94, *p* = .001, η^2^ = 1.075], showing that older adults were less accurate than young adults in using an non-egocentric strategy. Other interactions were not significant (all ps > .05).

Given that young and older adults showed differences in WAIS block design, digit span, virtual environment exposure, computer exposure, and practice trials, another repeated-measure ANOVA was performed with all of them controlled as covariates. We found the interactions were nonsignificant for all of these covariates, indicating these covariates had no significant impacts on age-related navigation performance. The main effect of age showed that older adults navigated significantly slower than young adults [F(1, 49) = 6.168, *p* = .017, η^2^ = .114]. The significant main effects of strategy revealed that non-egocentric strategy users completed the task with more rotations [F(1, 49) = 4.698, *p* = .035, η^2^ = .086] and more distance error [F(1, 49) = 6.643, *p* = .013, η^2^ = .108]. Moreover, the significant interactions between age and strategy revealed that non-egocentric older adults completed the star maze with more rotations [F(1, 49) = 6.072, p = .017, η^2^ = .107] and distance error [F(1, 49) = 7.372, *p* = .009, η^2^ = .116].

### Visuo-spatial ability in older adults

**Fig. 5:**
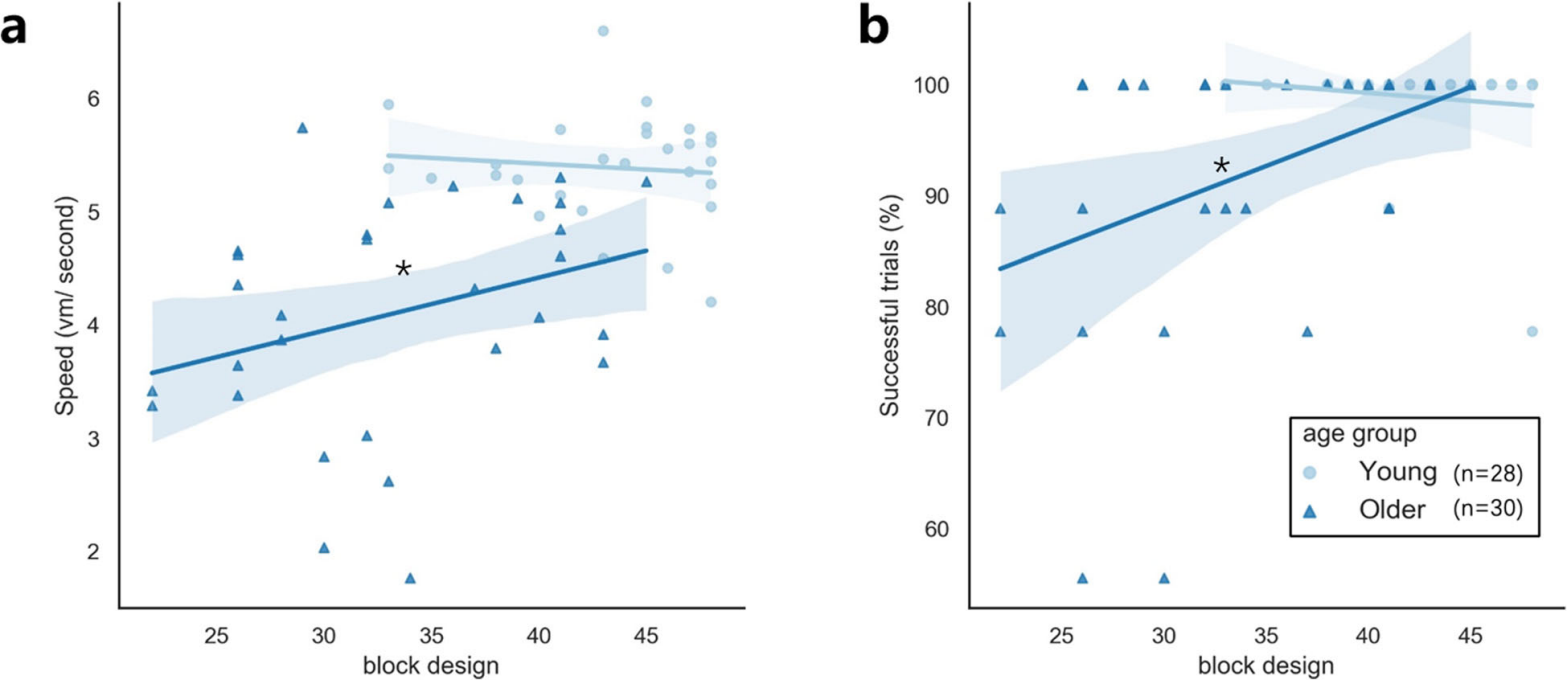
The correlations between WAIS block-design and navigation performances during the learning phase in older adults (*n* = 30) and young adults (*n* = 28). All the navigation performances are averaged across the 9th learning trials. The WAIS block design score is only correlated with speed (r = .370, *p* = .044) and percentage of successful trials (r = .417, *p* = .022) in older adults. All the other correlations are non-significant. Shadows indicate 95% confidence interval. ^*^p < .05

All the Pearson correlations in young adults (*n* = 28) were not significant (Table 4). For older adults, the WAIS block-design score was positively correlated with navigation speed (r = .370, *p* = .044) and the percentage of successful learning trails (r = .417, *p* = .022) (Table 4, Fig. 5), none of the other correlations were significant. These significant correlations suggest that better visuo-spatial ability in older adults was correlated with faster navigation speed and higher possibility to successfully complete the star maze.

**Table 4:** Correlations between navigation performance and cognitive tests in young and older adults

	WAIS block design		Forward digit span		Backward digit span	
	r	p	r	p	r	p
Young adults						
Speed (vm/ second)	−0.098	0.618	−0.064	0.747	−0.054	0.787
Rotation (°)	0.075	0.703	0.003	0.988	0.018	0.928
Distance error (%)	0.132	0.502	−0.167	0.397	0.015	0.938
Successful trials (%)	−0.144	0.465	0.021	0.915	−0.068	0.732
Older adults						
Speed (vm/ second)	0.370	0.044^*^	−0.123	0.519	0.207	0.272
Rotation (°)	−0.355	0.054	−0.030	0.876	−0.222	0.238
Distance error (%)	−0.267	0.154	−0.053	0.779	−0.190	0.313
Successful trials (%)	0.417	0.022^*^	−0.193	0.307	0.151	0.426

*WAIS* Wechsler Adult Intelligence Scale-IV; ^*^ *p* < .05

Furthermore, the independent sample t tests revealed that egocentric older adults scored higher on the WAIS-block design test than non-egocentric older adults, suggesting better visuo-spatial ability in egocentric older adults (Table 5). However, two groups did not differ in forward digit span (t = −.785, *p* = .457) and backward digit span (t = .041, *p* = .967). These results suggest that non-egocentric older adults have a specific deficit in visuo-spatial ability. In addition, the non-egocentric older adults showed more satisfaction and confidence about their memory (MMQ1) and reported less trouble about memory during the last fortnight (MMQ2), whereas the egocentric older adults preferred to adopt different memory strategies during daily life (MMQ3, though this result does not reach significant, the effect size is large), which may indicate a preserved ability to adopt or switch to an efficient strategy.

**Table 5:** Independent sample t tests between egocentric older adults and non-egocentric older adults

	Egocentric older adults (n = 7)		Non-egocentric older adults (n = 23)		t	Effect size (d)
	M	SD	M	SD		
WAIS block design	37.57	3.31	31.74	7.11	3.008^**^	1.05
Forward digit span	7.43	1.13	7.78	0.67	−0.785	−0.38
Backward digit span	5.29	1.38	5.26	1.39	0.041	0.02
MMQ-contentment	38.57	10.45	48.26	9.83	−2.252^*^	−0.96
MMQ-ability	41.79	9.21	52.78	10.12	−2.565^*^	−1.14
MMQ-strategy	39.29	14.73	27.41	13.48	1.999	0.84

*M* mean; *SD* standard deviation; *WAIS* Wechsler Adult Intelligence Scale-IV; *MMQ* Multifactorial Memory Questionnaire. ^*^ p < .05, ^**^ p < .01

## Discussion

In the current study, by using the virtual star maze task, we found older adults presented slower speed in virtual star maze navigation. Older adults adopting egocentric strategy performed as well as young adults, while older adults using non-egocentric strategy showed worse navigation performances than young adults. Moreover, better visuo-spatial ability was related with better navigation performance in older adults.

The current study found a selective deficit of older adults in spatial navigation speed, which was consistent with previous studies [3, 14, 37]. These results revealed age-related impairments of the navigation efficiency. Previous studies also found that older adults had slower reaction times, while their error rates were comparable to young adults in spatial representation task [53] and virtual navigation task [54]. A recent meta-analysis including 80 samples examined the age-related changes in spatial abilities. The results showed that the measures of response time yielded a larger effect size than the measures of accuracy, indicating that speed was a critical factor in spatial tasks, for instance, mental rotation and perception tasks [55]. This was consistent with the finding in the current study that revealed a significant effect of aging in navigation speed, but not in other performance. This specific impairment suggests that the navigation speed is also a sensitive index for aging in navigation.

The impairment of navigation speed may be the consequence of speed-accuracy trade-off [16]. It was established that older adults paid more attention to accuracy [56]. To successfully complete the task in the current study, older adults navigated at a significantly slower speed. Notably, even at the early stage of the learning phase, the percentage of older adults with successful navigation was comparable to young adults. Alternative explanation for the slower speed in older adults in virtual environment navigation might be the consequence of a poor perception of visual motion information [57]. Similarly, this speculation has been proved in vestibular perception [58] and direction perception [59, 60]. These deficits in primary perception in older adults may decrease the signal-to-noise ratios as well as the directional tuning, which may hamper higher-level cognitive processing [2].

Given the fact that the older adults showed less exposure to virtual environment and computers, it was worthwhile noting that, in current study, the average navigation speed across learning phase was significantly correlated with the experience of virtual environment (r = − 0.327, *p* = .012) and the experience of computers (r = − 0.332, *p* = .011), which were recorded in a 3-point scale. Meanwhile, the manual dexterity may also affect the navigation performance [61], which was not recorded in our study. Therefore, future studies need to test this possibility.

In the current study, we defined the egocentric strategy as a pure egocentric strategy ignoring the relationship between the landmarks and only relying on a series of body turns [14, 62]. The strategy discrimination was based on a hypothesis that the performance of egocentric navigators were not affected by the absence of distal landmarks, whereas non-egocentric strategies that relies heavily on the spatial relation of external landmarks were impaired in the probe trial [2, 9, 48]. The paired sample t test revealed that the navigation performance of the non-egocentric group in the probe trial was significantly worse than the 9th learning trial (Fig. 2), whereas this difference did not reach significance in the egocentric group. This dissociation was consistent with our hypothesis and supported the discriminative ability of the probe trial. We also examined these differences between 9th and probe trial in egocentric older adults and non-egocentric older adults, egocentric young adults and non-egocentric young adults separately. The results were similar and further verified the value of the star maze in differentiating strategies in both young and older adults.

A recent study demonstrated that the impairment in strategy use in older adults might further hinder their navigation performance [63]. For instance, older adults were impaired in strategy switching in the maze task [33]. Although previous studies revealed that the deterioration of navigation during aging might be specific to certain strategies [9, 64], the results were inconsistent. Some studies reported a specific impairment in allocentric navigation [14, 65], whereas others found a decline in egocentric strategy accuracy in older adults [16].

In current study, the main effect of strategy during learning phase was significant. In other words, although both egocentric and non-egocentric strategy can be used to complete the navigation task during the learning phase [6, 48], participants using the egocentric strategy performed much more accurate. Specifically, the superiority effects of egocentric strategy were only found in older adults. The results showed that non-egocentric older adults completed task with significantly more rotation and distance error, while egocentric older adults showed comparable navigation accuracy to both egocentric young adults and non-egocentric young adults during the learning phase. In current study, older adults used egocentric strategy less than young adults, indicating the inefficiency to use an appropriate strategy rather than a decline of egocentric strategy. Before the disappearance of distal landmarks in the probe trial, both egocentric strategy and non-egocentric strategy should be valid theoretically during the entire learning phase. As expected, the navigation performance of egocentric young adults and non-egocentric young adults did not show significant differences. The results demonstrated that older adults, but not young adults, were affected by the strategy use when the environment provided adequate landmarks for navigation.

The selective impairment of non-egocentric navigation in older adults may be attributed to the different cognitive loads of the navigation strategies [64]. To navigate in an egocentric way, participants were required to remember the time sequence of body turns [6]. For instance, in the current study, the time sequence was “straight – left – straight – right – straight – left – straight”, which can even be simplified as “left – right – left”. In contrast, non-egocentric strategy may be ambiguous and involve several strategies. Taking allocentric strategy as an example, navigation demands several cognitive functions, including encoding the spatial scene to form an aerial view, retrieving spatial imagery and route planning [66]. Previous studies have reported a greater activation of the frontal-parietal attentional control network in allocentric navigation than in egocentric navigation, which indicated an additional resource recruitment of allocentric strategy [64, 67]. Moreover, retrogenesis theory supports this selective impairment with another explanation that late-acquired abilities are more vulnerable to loss during aging [15]. According to this theory, egocentric strategy, as a more elementary representation, develops earlier, even before 5 years old, and is expected to be maintained during aging [16], whereas allocentric strategy is acquired by later school-age children [6] and is assumed to have deficits preceding egocentric strategy [13].

In the current study, we found the WAIS block-design was positively correlated with navigation performance in older adults, suggesting that visuo-spatial ability may play an essential role in the navigation of older adults, whereas the navigation of young adults may be relatively independent of visuo-spatial ability. The difference in environment encoding may explain why the navigation of young and older adults showed different extended dependence on visuo-spatial ability [68]. For example, previous studies reported that aging reduced the signal-to-noise ratios in the motion sensitive middle temporal area [69], and the difficulties in extracting the spatial information from optic flow may further impair the spatial updating, which is vital during navigation. In addition, older adults acquired less accurate memories for the spatial relationship of landmarks to complete the navigation task [37], and were less likely to use the geometric cues [32].

Moreover, we found no significant differences in MMSE and MMQ between the young and older adults, which excluded the possibility that the navigation performance impairment of older adults might be the consequence of a decline in general cognition or of amnesia. Even though, the difference of MMQ between egocentric and non-egocentric older adults might help to explain the strategy use of these two groups. In current study, the egocentric older adults may benefit from their ability to choose an appropriate strategy. Nevertheless, these results need be further verified.

The visuo-spatial ability may play an important role in the navigation of older adults. Specifically, in the current study, egocentric older adults showed significantly higher scores in visuo-spatial ability than non-egocentric older adults did. It has been reported that the visuo-spatial ability or the visuo-spatial component of working memory played a part role in encoding the environment information, which may underpin navigation performance [70]. Another real-world navigation study also revealed that participants with high visuo-spatial ability achieved higher accuracy and less time during navigation [71]. Moreover, visuo-spatial ability as well as navigation performance showed a clinical potential in diagnosis of MCI and AD [17, 19, 28]), which might provide a direction for future aging navigation studies.

There were several limitations in the current study. First, in the current paradigm, the probe trial could not subdivide the non-egocentric strategy into different strategies (e.g., allocentric and guidance strategy). Therefore, we hypothesized that all the participants kept using the same strategy during the entire experiment or, at least, they used an appropriate or an inappropriate strategy in the probe trial. In other words, we supposed that older adults who used an appropriate strategy learning the navigation task faster and better than older adults who used an inappropriate strategy. Second, the sample size may be not enough for a three-way ANOVA and the limited sample size (small number size in egocentric older adults’ group) restricted further analysis between two strategy groups in older adults. Therefore, our findings should be treated cautiously and further evidence need to be warranted.

## Conclusions

In the current study, older adults navigated the virtual star maze significantly more slowly than young adults but with comparable accuracy. Moreover, older adults using egocentric strategy performed as well as young adults, whereas older adults adopting non-egocentric strategy showed significantly lower navigation accuracy relative to young adults. The selective impairment was related with visuo-spatial ability in older adults. The current study provided direct evidence that navigational deficits could be markers for cognitive decline in older adults.

## Supplementary Information

**Additional file 1: Fig. S1.** The trajectories of two older adults in 9th learning trial and probe trial. The start point is at the bottom of the aerial view of the star maze and the destination is at the end of the top left alley.

## Data Availability

The datasets generated and analysed during the current study are not publicly available to protect the participants but are available from the corresponding author on reasonable request.

## Abbreviations

AD: Alzheimer’s disease
ANOVA: Analysis of Variance
MCI: Mild cognitive impairment
MMQ: Multifactorial Memory Questionnaire
MMSE: Mini-Mental State Examination
SD: Standard deviation
WAIS: Wechsler Adult Intelligence Scale
