# Supplementary material for: Age-related impairment of navigation and strategy in virtual star maze
Source: BMC Geriatr. 2021 Feb 5;21:108. doi: 10.1186/s12877-021-02034-y (PMC7866711; doi:10.1186/s12877-021-02034-y)
Supplement: Supplementary file 1 — Additional file 1: Fig. S1. The trajectories of two older adults in 9th learning trial and probe trial. The start point is at the bottom of the aerial view of the star maze and the destination is at the end of the top left alley. [file 12877_2021_2034_MOESM1_ESM.docx]

**
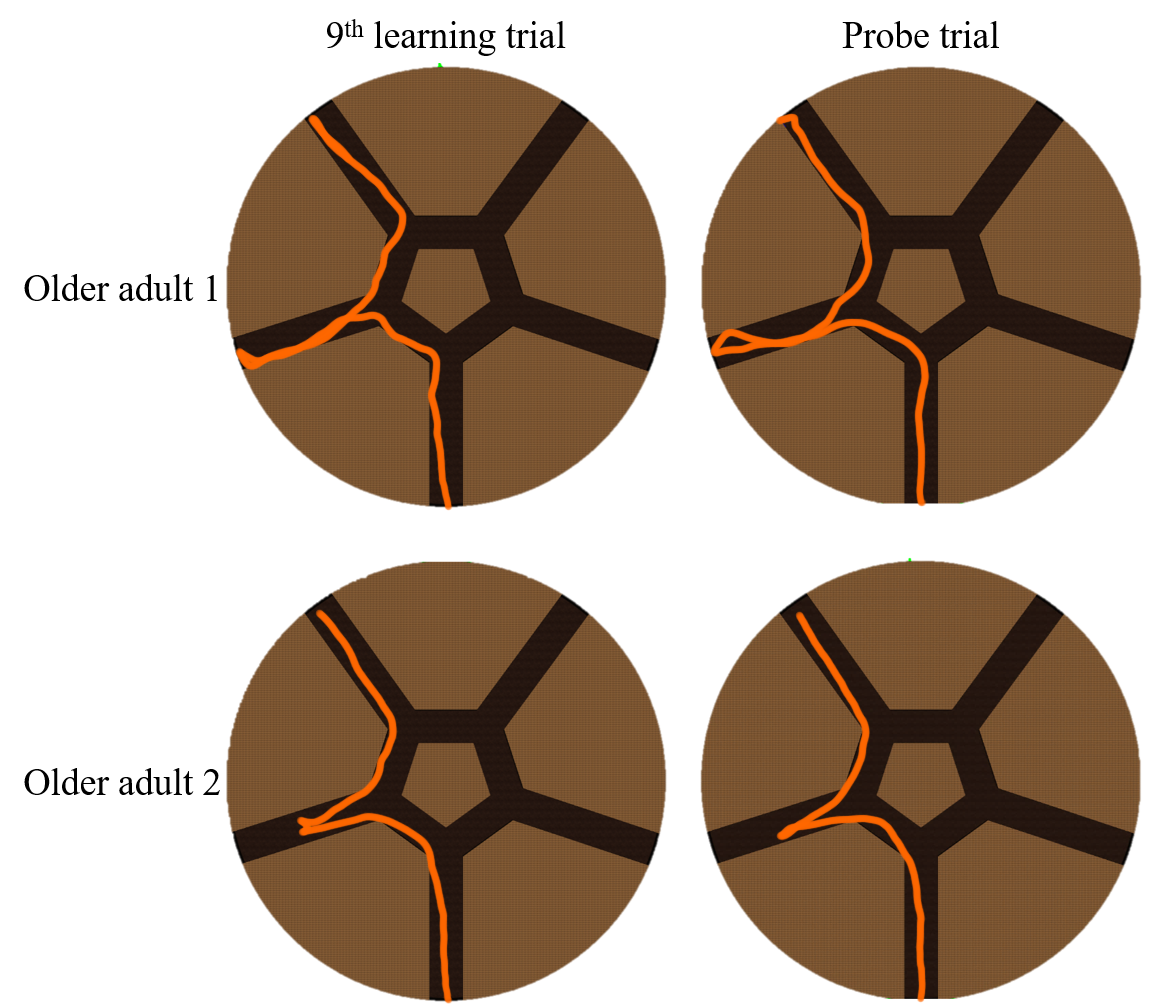
**

Supplementary Figure 1. The trajectories of two older adults in 9th learning trial and probe trial. The start point is at the bottom of the aerial view of the star maze and the destination is at the end of the top left alley.
